# Developing practical strategies to reduce addiction-related stigma and discrimination in public addiction treatment centers: a mixed-methods study protocol

**DOI:** 10.1186/s13722-024-00472-8

**Published:** 2024-05-16

**Authors:** Maryam Khazaee-Pool, Seyed Abolhassan Naghibi, Tahereh Pashaei, Koen Ponnet

**Affiliations:** 1 Department of Health Education and Promotion, School of Health, Health Sciences Research Center, Mazandaran University of Medical Sciences, Sari, Iran; 2 Department of Health Promotion and Education, School of Health, Kermanshah University of Medical Sciences, Kermanshah, Iran; 3 Department of Communication Sciences, imec-mict-Ghent University, Ghent, Belgium

**Keywords:** Strategy, Addiction-related stigma, Addiction-related discrimination, Addiction treatment centers, Mixed-methods study

## Abstract

**Background:**

People with substance use disorders (SUDs) have restricted engagement with health-care facilities and describe repeated experiences of stigma, discrimination, and mistreatment when receiving care at health-care and public addiction treatment centers (PATCs). The purpose of the current study is to design practical cultural-based strategies to reduce addiction-related stigma and discrimination at PATCs.

**Methods/design:**

The present study will use a mixed-methods design with an explanatory sequential approach. Phase 1 of the study will combine a cluster sampling technique combined with a cross-sectional survey of Patients with Substance Use Disorders (SUDs) in Mazandaran, Iran. A total of three hundred and sixty individuals with SUDs will be selected to assess their experiences of stigma and factors predicting stigma. Phase 2 will involve qualitative study aimed at exploring participants’ perceptions regarding the aspects and determinants of their stigma experience. The participants will include two groups: people with SUDs and staff/health-care providers at PATCs. Participants for Phase 2 will be purposively sampled from those involved in Phase 1.Qualitative data will be collected using in-depth semi-structured interviews and focus group discussions and analyzed using content analysis with a conventional approach. Phase 3 will focus on the development of new strategies to reduce the experiences of stigma among people with SUDs at PATCs. These strategies will be formulated based on the findings derived from the qualitative and quantitative data obtained in Phases 1 and 2, a comprehensive review of the literature, and expert opinions gathered using the nominal group technique.

**Discussion:**

This is one of the few studies conducted within the domain of stigma pertaining to individuals who use drugs within the context of Iranian culture employing a mixed-methods approach, this study aims to develop culturally sensitive strategies to reduce such problems from the perspective of Iranian people who use drugs. It is anticipated that the study will yield evidence-based insights and provide practical strategies to reduce the stigma and discrimination experienced by people who use drugs at PATCs. Such outcomes are important for informing policymaking and designing healthcare interventions tailored to the needs of individuals grappling with substance dependency.

## Introduction

Substance use disorders (SUDs) represent complex illnesses that disrupt brain activity and function resulting in significant personal and societal repercussions [1–4]. Recognizing the detrimental impact of SUD-related stigma, The National Institute on Drug Abuse has prioritized efforts to understand and diminish this stigma [5]. Research on mental illness stigma has consistently revealed its association with adverse outcomes, including exacerbated symptoms and impaired social functioning [6]. With the increasing prevalence of SUDs within the general population [1–4, 7] and the necessity to inform policymakers and allocate legislative resources effectively [8, 9], it becomes crucial to raise awareness about the stigma surrounding SUDs in society. Studies investigating SUD-related stigma have documented various forms of prejudice and discrimination experienced by people who use drugs, particularly from healthcare providers, which are correlated with detrimental health outcomes, including mental health disorders and compromised physical health [10–14].

Part of the stigmatization faced by healthcare providers stems from the inaction of public health leaders [15], while another part arises from the lack of training among healthcare workers in SUD treatment [16, 17], both of which contribute to inadequate implementation of effective remedies. Numerous studies have demonstrated the persistent and entrenched nature of stigma, often rooted in the misconception that drug addiction reflects a personal choice, indicating a lack of self-control and moral failure. Stigma and discrimination levels are notably high both within the general population and among professions that interact with individuals with SUDs, such as the healthcare industry. Some studies have demonstrated that stigma and prejudice harm SUD patients’ health and cause delays in the delivery of high-quality care in venues for public addiction treatment. Individuals with SUDs frequently encounter stigma and discrimination across all levels of care at public addiction treatment centers (PATCs) [17–22].

The World Health Organization is working with several countries to train medical professionals in screening, brief intervention, and referral to treatment (SBIRT) [23–27]. SBIRT is a treatment strategy that encourages all medical professionals to identify patients who are taking drugs at statistically dangerous levels, provide brief interventions to promote drug use reduction, and then refer patients who meet criteria for drug use or addiction for more intensive treatments. According to some studies, screening and brief interventions (SBI) have the greatest effect on reducing the use of psychoactive substances [20, 23, 26, and 28]. SBI is a simple, quick advisory intervention that stresses several types of specific behavior. It may be used by professionals in a variety of situations [29].

Unfortunately, societal acceptability of evidence-based initiatives does not always come easily [30]. The allocation of healthcare interventions is influenced by various factors, including the novelty of characteristics, healthcare worker attitudes, and the stigma associated with a health condition. Research has consistently demonstrated that negative attitudes among healthcare professionals can impede the adoption of innovative practices, the quality of services provided, and clients’ adherence to preventive and therapeutic measures [31–36]. Therefore, education and training programs should prioritize the modification of attitudes and beliefs among healthcare providers to promote the uptake of SBI for drug addiction [37, 38].

Research in health has linked stigma from service providers at care or treatment centers with poor utilization of preventive programs and reduced accessibility for stigmatized individuals to access effective interventions [39, 40]. Efforts to mitigate stigmatization are underway, particularly for individuals living with mental health conditions [40, 41]. Studies have identified three main approaches: (i) providing educational interventions to dispel misconceptions about mental illnesses, (ii) facilitating interactions between individuals with mental illnesses and the community to challenge community attitudes, and (iii) exposing stigmatizing beliefs and behaviors in the hope of eliciting public condemnation and reducing their acceptance [41–43]. Although anti-stigma strategies are sometimes inaccessible or unproven, the aforementioned techniques aim to change community perceptions of people facing such circumstances [39, 41].

To reduce the stigma associated with mental illness, several national and international strategies have been developed, and the range of programs continues to expand. However, stigma and discrimination against individuals with SUDs remain poorly understood [44, 45]. Moreover, there has been limited research investigating the creation and execution of practices or interventions aimed at reducing SUD-related stigma and discrimination among people who use drugs by PHC professionals [46–50]. When developing anti-stigma strategies, it is essential to consider the cultural norms and different behaviors of specific groups, including healthcare professionals, youth, police, and policymakers [14, 38, 40, 45, 49].

For many years, stigma related to SUDs has posed challenges in Iran [51–53]. One of the most significant obstacles to improving the well-being and health of individuals with SUDs is the stigmatization and discrimination they face within the healthcare system [52, 54]. This results in disparities in healthcare facilities, including limited availability, accessibility, and quality of services for individuals with SUDs [54]. Stigmatization negatively impacts help-seeking behavior from official healthcare facilities, leading to poorer outcomes and perpetuating the misconception that SUDs are untreatable. individuals with SUDs may be more prone to engaging in unhealthy behaviors, refusing treatment, non-compliance with prescription instructions, weakened immune systems, and experiencing adverse consequences [55].

Comprehensive plans for the promotion, prevention, treatment, and recovery of individuals with substance use disorders (SUDs) should consider numerous socioeconomic variables. Adopting a “health-in-all policies” approach is crucial in addressing these challenges. Strategies to increase access to treatment and reduce stigma and discrimination towards individuals with SUDs may involve integrating SUD care and fostering collaboration between primary care clinicians and other healthcare providers [22, 38–40, 53]. International efforts to combat addiction-related stigma have emphasized the importance of lowering barriers to a variety of health treatments for individuals with SUDs. Despite this emphasis and the widespread consensus that reducing stigma associated with SUDs is important, progress in this area has been slow [40, 49, 56–58]. While strategies to reduce SUD-related stigma have gained traction in Western industrial nations in recent years [59, 60]. They remain largely absent from national and government policies, information, and healthcare plans in many parts of the world [40, 42, 44, 53, 58, 61].

Longitudinal data on behavior changes in response to stigma and discrimination related to SUDs in Iran are lacking, making it challenging to develop effective strategies to reduce such stigma, especially in PATCs. The most widely recognized solutions are those that are acceptable, suitable, and adaptable across cultural contexts. Further research and needs assessments are required to identify additional strategies for addressing addiction-related stigma [42, 47, 56]. To address the stigma associated with addiction, it is necessary to study the effectiveness and feasibility of stigma-reducing interventions [55, 58, 62].

In Iran, as in many other countries, there is a lack of comprehensive strategies aimed at reducing stigma related to SUDs. Additionally, there is a dearth of studies providing practical strategies, both quantitative and qualitative, to address addiction-related stigma and discrimination specifically within PATCs for individuals with SUDs in Iran. Mixed-method analyses focusing on this issue are also lacking. While there have been some studies conducted in Iran to explore stigma toward individuals with SUDs, none have offered strategies or methods to mitigate stigma within public treatment settings. Although limited, existing data from small-scale qualitative studies in Iranian healthcare settings indicate the prevalence of discriminatory attitudes toward people with SUDs, manifesting as care refusal, substandard care, excessive precautions, physical distancing, humiliation, and blame [30, 51, 52, 55, 62–64].

Iran’s unique cultural characteristics [65] including demographic factors [66], cultural norms [67], ethnic identity [68], social customs, traditions, peer relationships, and poverty [69] shape the societal landscape and perceptions surrounding behaviors, including those related to SUDs. Consequently, addressing addiction-related stigma and its impact on individuals who use drugs in Iran requires sensitivity to these cultural nuances [64, 70]. In Iran, SUDs are not solely viewed as medical issues but also as a socio-cultural problem. This perspective can lead to delays in treatment and pose significant challenges for patients and their families. Consequently, reducing stigma and discrimination associated with the rising prevalence of addiction among Iranians has been identified as a pressing priority within the healthcare system [70].

In Iranian society, plays a significant role in shaping perceptions and experiences of SUD across various demographic groups, including differences related to age, gender, socioeconomic status, and education level [64]. Research in Iran has extensively explored how cultural influences manifest in SUDs, examining factors such as demographic characteristics, regional prevalence patterns, gender dynamics, religious beliefs, and the stigma associated with drug use. These studies highlight the complex interplay between cultural norms, individual behaviors, and societal attitudes toward SUDs within the Iranian context [66–68, 71–80].

Of course, the stigma surrounding drug addiction in Iran exhibits variations based on factors such as gender, the specific type of drug used, and residential location [81, 82]. Interestingly, a study examining literary works in Iran reveals a historical acceptance of opium as a medicinal remedy by prominent Iranian poets. Opium has been portrayed positively, with references to its purported benefits such as regulating blood pressure and relieving pain [83]. This cultural perspective reflects a nuanced view of drug consumption in Iranian society, indicating that stigma surrounding certain drugs may not be uniform. Rather, stigma appears to evolve dynamically within social contexts, presenting new challenges that may differ from those associated with more entrenched forms of stigma.

Although previous qualitative studies have provided valuable insights into the experiences of individuals with SUD interacting with healthcare professionals, our understanding of SUD-related stigma within the Iranian healthcare system remains limited. A comprehensive, multiphase study employing a mixed-methods approach is needed to systematically assess the experiences of Iranian people who use drugs regarding stigma and to develop evidence-based guidelines and strategies for reducing stigma and discrimination against individuals with SUDs at PATCs. The importance and the impact of stigma and discrimination related to SUDs within Iranian culture as well as the influence of cultural differences on patients’ healthcare-seeking attitudes and the support services provided by the healthcare system, form the foundation of this mixed-method study. Given these considerations, it is imperative to address cultural factors associated with substance use disorders and the stigma stemming from substance consumption in Iranian society. This is because the cultural, economic, and social variations across different societies warrant an examination of human experiences within each unique cultural context. Therefore, the aim of this study is to explore comprehensive and culturally sensitive strategies in order to reduce addiction-related stigma and discrimination at PATCs.

## The study aims

This mixed-methods study aims to identify strategies to reduce stigma and discrimination against Iranian people who use drugs at PATCs. The specific objectives of the study can be categorized into three phases as follows:

### Phase 1

1. To measure the perceived stigma score among people with substance use disorders (SUDs) who were referred to PATCs in Mazandaran, Iran.
2. To evaluate professionals’ attitudes towards people with SUDs receiving treatment at PATCs in Mazandaran, Iran.
3. To measure the social distance score towards people with substance use disorders seeking treatment at PATCs in Mazandaran, Iran.
4. To examine the relationship between socio-demographic characteristics and perceived stigma among individuals with substance use disorders.
5. To investigate the relationship between perceived stigma, social distance, and professionals’ attitudes toward people with SUDs.

### Phase 2

6. To explore the perspectives and experiences of people who use drugs concerning the various aspects and determinants of stigma and discrimination stemming from the community, healthcare centers, or PATCs due to drug use.
7. To examine healthcare providers’ perspectives on stigma against individuals who use drugs.

### Phase 3

8. To develop evidence-based cultural strategies aimed at diminishing stigma and discrimination at PATCs against Iranian people who use drugs faced health challenges.

## Methods/design

### Study design

This study will employ a mixed-methods technique with an explanatory sequential approach for data collection and analysis. Grounded in pragmatic principles and logic, the mixed-methods paradigm combines quantitative and qualitative methodologies to provide a comprehensive understanding of the research questions. In this methodology, the researcher first gathers quantitative data to identify patterns or trends requiring further exploration. Subsequently, qualitative data are collected from individuals who can offer insights to enhance the understanding and interpretation of the quantitative findings [84]. According to this paradigm, merging qualitative and quantitative methods results in a deeper comprehension of the issue [85, 86].

**Fig. 1:**
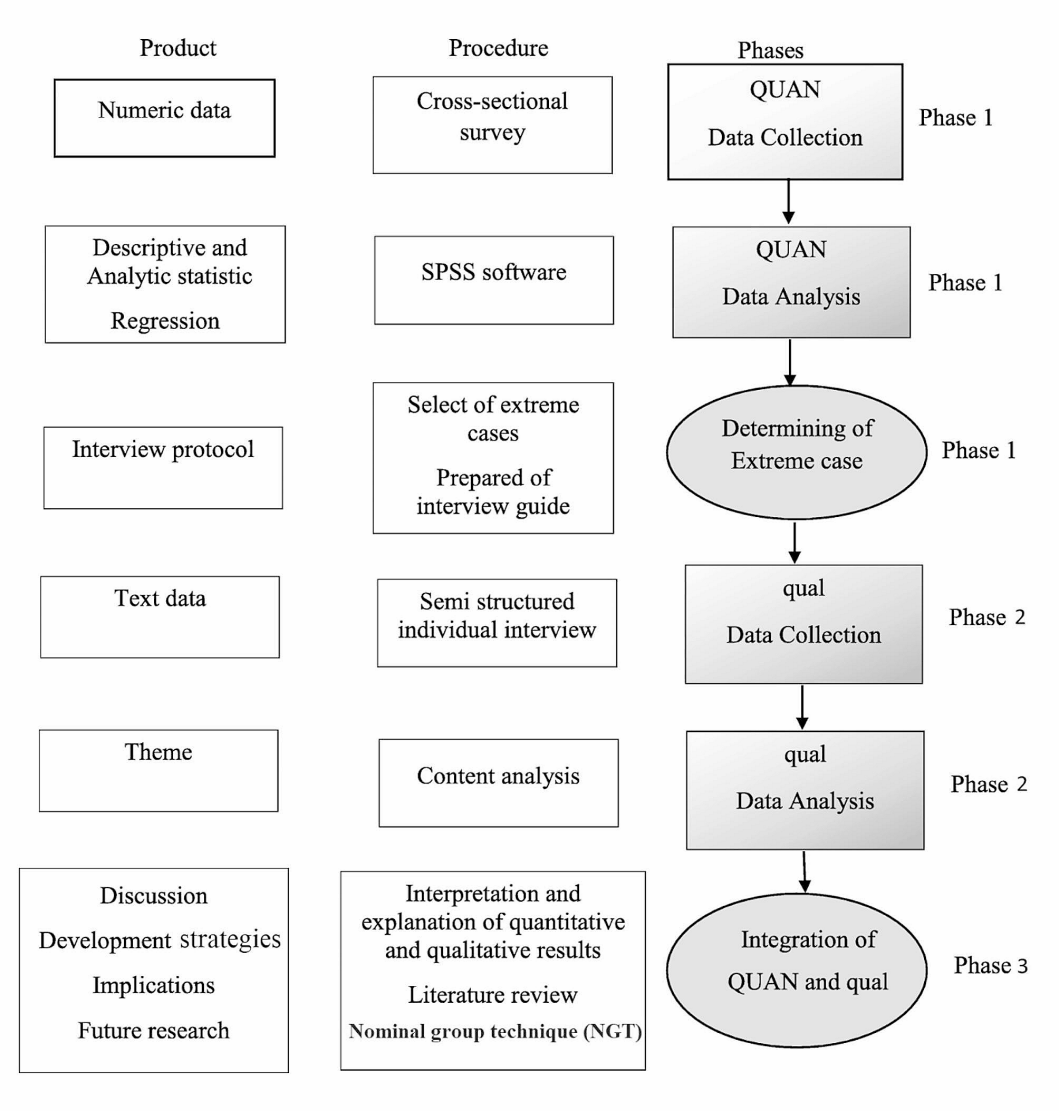
Study visual diagram

This study will be conducted in three phases. The first phase will be a quantitative study, during which, quantitative data will be gathered. The second phase of this project will be a more detailed exploratory qualitative study of participants’ experiences regarding SUD-related stigma toward and discrimination against people who use drugs at PATCs. At the end of the second phase, the qualitative and quantitative findings will be integrated. The third phase of the study will involve the development of evidence-based and culturally sensitive strategies based on a literature review, the results of Phases 1 and 2, and experts’ opinions using the nominal group technique (NGT) (Fig. 1). Full explanations of each part of the study are provided below.

### Phase 1: quantitative study

The quantitative phase will be a descriptive-analytic cross-sectional study conducted among Iranian people with SUDs living in Mazandaran, Iran. In this phase, we will assess perceived stigma experiences and their relationship with social distance, perceived dangerousness, experts’ discrimination or acceptance, and sociodemographic characteristics among the participants. The target population will consist of people who are referred to PATCs in Mazandaran, Iran. The Perceived Stigma of Addiction Scale (PSAS), Health Professionals’ Attitude Towards Substance Abusers Scale (HPA-SAS), and Social Distance Scale (SDS) will be used. These scales will be validated for use among Iranian people.

### Sample size and sampling method

There is no shortage of research on stigma toward and discrimination against people with SUDs at PATCs and other health-care settings in Iran. Therefore, the sample size is calculated based on Matsumoto’s study [87]. Following Matsumoto et al. [87], the calculated sample size is 240, based on the largest standard deviation related to the sub dimension of stigma (SD = 12.39), with a precision (d) of 0.05 around the mean (m = 35.01), and α = 0.05. In most cases, the design effect’s numerical value is about 1.5–2. In this study, we will apply 1.5, and the final sample size will be increased to 360 substance users, based on cluster sampling.

For this project, fifteen PATCs in Mazandaran will be selected. A cluster sampling method will be employed, with each cluster comprising a comparable number of respondents. Mazandaran will be divided into three areas (west, central, and east). All PATCs within these areas will be enumerated, and five PATCs will be randomly chosen from each area. Individuals with SUDs who are referred to the PATCs will be invited to participate in the project.

The participants will be offered comprehensive explanations of the goals and methods of the research. The sociodemographic questions, the PSAS, HPA-SAS, and SDS will be administered in a “quiet setting” [questionnaire will be presented while maintaining patient privacy] by a research group member and then collected in person. The investigator will fill out the scales to ensure that the same data collection method is used for all individuals. Informed consent will be obtained from the individuals prior to the data collection.

### Inclusion criteria

Individuals will be eligible for the current project if they are adults (aged 20 years or older), reside in Mazandaran province, have a history of any kind of substance use, and have no severe mental difficulties that prevent them from answering the items in the questionnaires.

### Exclusion criteria

The exclusion criteria for participants will be: having a mental disability, having psychiatry history like active bipolar disease, depression with psychosis, or schizophrenia, being deaf or mute, showing unwillingness to continue participating in the study, and not fully completing the questionnaires.

### Questionnaires and data collection

Quantitative data will be collected utilizing sociodemographic variables and the PSAS, HPA-SAS, and SDS scales. The sociodemographic section will include questions on age, gender, occupation, duration of employment, and education. The PSAS comprises eight items to measure the perceived stigma towards individuals with substance use disorders. Initially developed and validated among patients undergoing treatment for substance use–related issues in the United States [88]. he items were adapted from a study conducted by Link and colleagues on perceived discrimination-devaluation processes, Content validity was established through review by stigma professionals in the substance use field the PSAS was related to adopted shame, self-concealment, adopted stigma, and depression [89]. The PSAS employs a four-point Likert scale ranging from “strongly disagree” to “strongly agree” for participants to rate their agreement or disagreement with each item. Scores range from 8 to 32, with higher scores indicating greater perceived stigma. The PSAS has demonstrated good reliability, with a Cronbach’s alpha of 0.71 and a reliability coefficient of 0.79 based on the test-retest method in American society [88]. In an Iranian study, the reliability of the PSAS was found to be 0.85, with a test-retest correlation coefficient of 0.81 [90].

The HPA-SAS consists of 10 items, with questions addressing the attitudes and/or views of health professionals toward people with SUD, their knowledge of addiction, and their training in substance use. The constructs of attitudes will focus on discrimination and acceptance towards people who use drugs. The HPA-SAS was developed utilizing a Likert scale format, with each item offering four response options: (1) strongly disagree (2), disagree (3), agree, and (4) strongly agree, resulting in total scores ranging from 10 to 40. The validity and reliability of the HPA-SAS were established through research conducted by a team of psychological counseling and medical care professionals. The overall Cronbach’s alpha of the original HPA-SAS has been reported as 0.79 [91]. In this study, the validity and reliability of the questionnaire were assessed prior to data collection with a sample of Iranian people who use drugs. The overall Cronbach’s alpha of the HPA-SAS was found to be 0.76, and the test–retest correlation coefficient of this scale was 0.74.

The seven-item SDS, which was created by Bogardus et al. (1925) [92] and then modified by Link et al. (1987) [89], measures the social distance that interviewees wish to keep toward a person with a particular condition (diverse social, ethnic, or racial backgrounds). This scale focuses on respondents’ willingness to engage in a relationship with someone who is dependent on illegal substances. In particular, it measures people’s willingness to take part in a variety of social contacts with a particular group. The SDS consists of seven items presented as multiple-choice questions, which assess social distance by probing the respondent’s willingness to engage in various social interactions with stigmatized individuals: These interactions include scenarios such as being a sub-lessee, neighbor, co-worker, spouse of a family member, caretaker of one’s child, and member of the same social group. Participants will be asked to rate their level of willingness or unwillingness for each item using a four-point Likert scale with the following options: (0) definitely willing [1], willing [2], unwilling, and [3] definitely unwilling. The total score ranges from 0 to 21; scores higher than the mean identify higher social distance. The overall Cronbach’s alpha of the original SDS is 0.75 [89]. The Iranian version of the SDS has found to have a Cronbach’s alpha value of 0.92. The test–retest reliability coefficient for the SDS was 0.89, and the content validity coefficient was 0.75 [90].

### Data analysis

The data from the first phase of the study will be analyzed using SPSS Statistics Version 26.0 for Windows (IBM Inc., Armonk, NY, USA). In the cross-sectional phase, descriptive statistics will be applied to describe the sociodemographic factors and perceived stigma of addiction, experts’ attitudes toward people with SUD, and social distance. Univariate analytical statistics will be used to test the correlation between the sociodemographic variables and perceived stigma, experts’ attitudes toward people with SUD, and social distance. Variables with a correlation of *p* < 0.1 in the univariate analysis will be included in the multivariable logistic model. All statistical tests will be two-tailed, and a *p*-value < 0.05 will be considered statistically significant. To ensure data quality during this phase of the study, measures such as double data entry and range checks for data values will be implemented.

### Phase 2: qualitative study

In Phase 2, an exploratory qualitative study will be conducted utilizing a conventional content analysis method to explore the experiences of people who use drugs regarding stigma and discrimination stemming from the community, health-care centers, or PATCs as a result of drug use. Additionally, this phase will aim to gain insight into healthcare providers’ perspectives on stigma against people who use drugs in greater detail. Given the objectives of the project’s qualitative phase, employing this method will enable the investigator to gain a comprehensive understanding of the situation, facilitating the clarification of the impact of stigma and discrimination on Iranian people who use drugs at PATCs.

### Participants and sampling method

A purposive sampling approach will be used in the second phase of the study. The target population will consist of two groups of people, namely, those who have experienced drug use and staff members at PATCs. The first group of participants (people who use drugs) will be selected from those willing to participate in the quantitative phase of the study and will be based on the mean total score of the stigma experience, which will be collected in Phase 1 of the study. People with 10% upper and lower stigma experience scores will be selected as extreme cases, and will be retained for the next phase. We will seek to interview people with either a stigma or discrimination experience in order to collect more comprehensive information about their stigma experiences and its related factors. Efforts will be made to have variety in terms of gender, level of education, religion, age, socioeconomic situation, and the use of different types of drugs.

The second group of participants will consist of health-care workers and providers at PATCs. This sample will include agents from (i) PATC management, (ii) clinical and medical teams, (iii) health-care program teams and (iv)others according to the setting (e.g., finance). Health-care workers will be enlisted using purposive sampling methods. Four of them will be contacted through education programs with a specific focus on staff involved in drug treatment. The retained persons will be invited to register, and a member of the research team will be in touch to schedule an interview. Health-care workers in specific treatment centers will also receive direct invitations from the investigation team.

Data analysis will commence after the first interview, focusing on elucidating the intricacies and interactions among key concepts and categories derived from the exploration of the primary data. Consequently, the selection of participants will persist until theoretical saturation is achieved, ensuring a comprehensive understanding of the relationships between the study concepts and components [93]. In the current study, sampling will continue until the investigator determines that no further data can be garnered through data analysis and coding, signifying theoretical saturation. However, it is recommended by experts that a minimum of 12 participants be interviewed for a qualitative study to ensure a robust and comprehensive analysis [94].

### Data collection

Data will be collected by two methods: in-depth interviews with individuals with SUDs and focus group discussions with PATC staff members.

#### Semi-structured, in-depth interviews

Individual, in-depth, semi-structured interviews featuring open-ended questions will be employed to gather data. These interviews will focus on exploring participants’ perspectives and experiences related to stigma and discrimination against individuals with (SUDs within healthcare settings. The target group for this part of the study will consist of people who use drugs who have been referred to PATCs in Mazandaran, Iran. Before the qualitative phase of the study, the interview protocol questions will be prepared based on the results of the first phase of the study as well as the literature review. Interviews will be held in locations, such as clinics, where respondents will feel safe and relaxed. All individual in-depth interviews will be recorded using a digital tape recorder after the applicant’s permission. In addition to the audio recordings, the interviewer will take notes. If participants decline to be audio-recorded, only notes will be employed for data gathering. Furthermore, non-verbal cues, such as facial expressions, tone of voice, and the respondents’ state, will also be noted by the interviewer, together with the date and place of the interview.

All interviews will be conducted by the first author of this study, who is familiar with qualitative research methods and the topic, and who has conducted similar studies on addiction,. Participants will be encouraged to discuss their experiences related to strategies to reduce addiction-related stigma and discrimination in public addiction treatment centers. Further, they will be encouraged to discuss sociocultural and ecological components that might have had an effect on the level of using these strategies in this regard.

The interviews will be focused on the following three main questions:

- How was the experience with stigma toward and discrimination in health-care settings?
- What strategy and procedure have they applied to reduce and cope with stigma and discrimination in health-care settings?
- How have the strategies and procedures affected their coping strategies in this regard?

Based on the responses to these questions, follow-up questions will be asked. After each question, participants will be invited to explain more thoroughly their answer, by probing questions such as “What do you mean?” or “can you explain this more”.

Interviews will be performed during a single meeting with each participant and is estimated to last between 40 and 60 min, although this can differ slightly based on the experiences of each participant. The investigator will start with explaining the significance of the study in order to gain their confidence. All interview questions will be reviewed after the first interview, and all interviews will be taped. Data collection will be continued until saturation is reached.

#### Focus group discussions

Following semi-structured interviews, the principal researcher (first author), who is an expert in qualitative studies, an expert in qualitative studies, will conduct focus group discussions with staff members at Patients with Substance Use Disorders Treatment Centers (PATCs), which comprise the second target group of this phase of the study. These focus group discussions aim to validate the emerging themes from the individual interviews and gain deeper insights into the identified themes. The focus group discussions will be guided by the two main research questions: (i) What is providers’ understanding of stigma towards and discrimination against persons with SUDs? and [2] What are the providers’ opinions regarding a response to stigma and discrimination? Furthermore, more detailed investigative questions will be incorporated, such as: What types of SUDs do your clients typically present with? Are there any other community-level factors that could influence experiences of stigma and discrimination against individuals with SUDs?

### Data analysis

Immediately following data collection, the coding process will be initiated, and the data will be analyzed. The main themes will be identified using a conventional content analysis method of Graneheim and Lundman [95], in which themes and subthemes are identified to reveal participants’ perceptions and experiences toward stigma and discrimination against Iranian people who use drugs at PATCs. This process will employ inductive reasoning, which introduces concepts and categories via a detailed exploration of the data by the researcher.

In Graneheim and Lundman’s method, qualitative content analysis addresses the obvious content of an interview, along with explanations of content that can be construed or added from the interview but are not obviously detailed in the transcript [95]. Further, coding classifications are derived directly from the transcription data. Without laying on preset themes or prior theoretical opinions to categorize extracted codes from interviews, the conventional content analysis method is a suitable technique for advancing coding categorizations from the raw interview transcripts.

In this method, data analysis begins with a comprehensive reading of the entire text to ensure a thorough understanding. Subsequently, the text is examined word by word to extract codes, initially identifying specific words that may encapsulate the main concepts. These texts are derived from notes documenting the initial opinions of the interviewees and the preliminary analysis conducted. Codes that are indicative of more than one main thought are tagged and then categorized based on their dissimilarities and similarities. The greatest benefit of a conventional content analysis is attaining data directly from the study without imposing preplanned and defined categories, themes, or theories. However, one problem with this kind of analysis is that it interjects with other qualitative methods (i.e., grounded theory or phenomenology). While these approaches share similarities with initial analysis, they are emphasized for their relevance to theory advancement. Additionally, they hold significance for model development. To evaluate the trustworthiness of the results in this phase of the study, four criteria —reliability, portability, credibility, and verifiability— will be employed [96]. MAXQDA software will be used for data processing.

### Phase three: integration of quantitative and qualitative data and the development of strategies

In this phase, cultural evidence-based strategies aimed at reducing stigma and discrimination associated with substance use of Iranian people at PATCs will be developed This will involve integrating insights from the literature review, the findings of the preceding study phases, and input from experts. The target group for this aspect of the study will comprise PATC experts residing in Mazandaran, Iran.

Upon completion of the second phase of the study, the quantitative and qualitative results will be merged to glean additional insights that will inform the design and implementation of appropriate strategies to mitigate stigma and discrimination against individuals with SUDs at PATCs. Three techniques can be employed to integrate the quantitative and qualitative findings: combining the data into a discussion, utilizing a matrix for combination, or employing a side-by-side display and transformation. n this study, the data will be combined into a discussion format. Some researchers often commence this approach with a section outlining the quantitative findings, followed by a section detailing the qualitative findings. Alternatively, researchers may present the quantitative findings while substantiating claims with quotes extracted from them. Another less common technique involves initially presenting the quantitative results and subsequently confirming and validating them with descriptive qualitative findings [97, 98].

To develop strategies for reducing stigma and discrimination against people who use drugs at PATCs, the research team will start with formulating guidelines after a comprehensive review of the available literature. Systematic review and interventional studies will be conducted to find approaches. The search will encompass English-language databases (including Cochrane Library, APA PsycNET, MEDLINE, Web of Science, Embase, Scopus, ProQuest) as well as Persian databases (such as Magiran, Irandoc, SID, and Barakat). No restrictions will be imposed, particularly with regard to publication dates, to ensure comprehensive coverage of relevant studies. A uniform search strategy will be applied across all databases, utilizing the intersection of three fields via the Boolean AND operator. To define search terms, the Medical Subject Headings (MeSH) dictionary will be referenced. Upon identification of relevant documents, their quality will be assessed using the GRADE approach, followed by evidence analysis. Insights gleaned from the literature review will also be incorporated. Subsequently, the recommended strategies developed will be offered to Nominal Group Technique (NGT) experts.

NGT will be applied will be employed to devise and implement effective strategies aimed at diminishing stigma and discrimination against individuals with SUDs at PATCs. NGT is a structured, group-based method utilized to achieve consensus. Participants are encouraged to independently generate viewpoints based on predetermined and organized questions facilitated by a moderator [99]. To initiate the NGT process, primary strategies will be extracted from the findings of the first and second phases of the study, in addition to insights gathered from a literature review and examination of relevant rules and regulations A meeting will then be held with the experts who must meet the inclusion criteria of being residents of Mazandaran, Iran, possessing a minimum of one year of relevant work experience, having comprehensive familiarity with Iranian culture and customs, and being employed in a clinic associated with the treatment of people who use drugs. During this meeting, specialists will be invited to share their opinions on the developed strategies in relation to the key study questions, with all ideas and suggestions being meticulously recorded. Subsequently, these suggestions will be organized and prioritized to formulate consensus-driven strategies for effectively reducing stigma and discrimination against Iranian individuals with SUDs.

### Ethical approval

The Ethics Committee of the Mazandaran University of Medical Sciences in Mazandaran, Sari, Iran, has approved the protocol for the present study [code number: IR.MAZUMS.REC.1401.192]. Informed written consent will be obtained from all participants during the quantitative and qualitative stages. Participants will be assured of the confidentiality of their data and identities. Additionally, they will be informed that they have the right to withdraw from the project at any phase of the intervention, and that their decision to refuse participation at any time will not impact or alter the quality of services provided to them.

## Results

The study is still ongoing, and no results have yet been generated. We will wait until the completion of our first data collection before disseminating any findings.

## Discussion

This article outlines the protocol for a mixed-method study aimed at identifying and formulating appropriate strategies to mitigate addiction-related stigma and discrimination at PATCs. The study will offer comprehensive insights into the stigma encountered by a cohort of Iranian people who use drugs and the factors influencing their experiences. The findings of this study will be utilized to develop and implement culturally tailored strategies geared towards reducing stigma and discrimination associated with substance use among Iranian people who use drugs attending PATCs.

While stigma and discrimination linked with drug addiction is a global concern, their nature and expression are contingent upon the religious, social, and cultural frameworks prevalent in various societies. Operating as a multilevel phenomenon, stigma arises when harm resulting from unfavorable status, labeling, or discrimination transpires within a power structure that perpetuates and reinforces social inequalities among those labeled [100]. Stigma toward substance use can profoundly impact an individual’s social and personal connections, often resulting in feelings of worthlessness. Such stigma may provoke negative responses and behaviors from various organizations and individuals towards the affected person [101, 102]. These behaviors can impede access to treatment for individuals with substance use disorders. Moreover, they contribute to social, financial, and health discrimination within these communities, fostering the perception that individuals with SUDs are undeserving of the opportunity to address their condition [103].

Stigma significantly impacts the spectrum of care for individuals with SUDs, influencing aspects such as treatment seeking, preference, maintenance, and adherence, consequently leading to poorer health outcomes within this population or ever, stigma may exacerbate disparities in accessing medical and health services, as individuals with SUDs may be hesitant to pursue and adhere to health-oriented measures [104].

Studies evaluating the stigma experiences of persons with SUDs are mainly qualitative in nature [21, 52, 62, 98, 105, 106]. The present study will be one of the few studies addressing addiction-related stigma in Iran that applies a mixed-methods technique to identify suitable strategies to reduce addiction-related stigma and discrimination at PATCs from the perspective of Iranian people who use drugs. It is expected that the current work, by using quantitative and qualitative methods, will offer valid data regarding suitable cultural strategies to reduce stigma against persons with SUDs at health-care and treatment centers.

The findings of the current study hold potential significance for healthcare specialists and policymakers shedding light on the pivotal role of cultural strategies in mitigating stigma against individuals with SUDs within healthcare and treatment settings employing a culturally sensitive approach Furthermore, the study aims to elucidate the needs of individuals with SUDs and provide insights into the factors influencing addiction-related stigma that require attention. Effective strategies emerging from this research may encompass interventions geared towards enhancing the health outcomes of Iranian people who use drugs and their families, as well as those from other nationalities and countries sharing similar cultural contexts with Iran. Additionally, the study’s findings are anticipated to inform stigma-reduction education and healthcare support initiatives tailored to the Iranian population, underpinned by a culture-based approach.

### Potential strengths of the study

This study has several advantages. The results will potentially fill some of the gaps in research on people with SUDs who encounter stigma and discrimination at PATCs thus holding significant clinical implications. By employing a mixed-methods approach, this study facilitates the integration of diverse approaches and methodologies. The collection of both qualitative and quantitative data will provide a comprehensive understanding of. People who use drugs’ experiences of stigma and discrimination at PATCs. Moreover, the qualitative component of the study involves various participants directly or indirectly associated with this phenomenon, including individuals with SUDs and staff/clinicians. Conducting interviews with substance users and clinicians will enable a deeper understanding of how the phenomenon is perceived by those directly affected by stigma/discrimination, as well as by individuals closely involved in the patients’ daily lives and clinicians, who play a crucial role in both the phenomenon and its treatment.

### Potential limitations of the study

The researchers acknowledge several limitations in the current study although the developed strategies will be evaluated upon achievement to ascertain their suitability and effectiveness, detailed descriptions will be necessary to design appropriate interventions and allow for generalization in similar contexts. One limitation is related to the sampling, which will be conducted in only one province in Iran. To mitigate this weakness, we will try to use maximum variation in the study phases. Another limitation is the possibility that the participants will not cooperate and drop out before the end of the study. Additionally, the scarcity of research and literature reviews regarding the stigma experienced by this population at PATCs poses a challenge. Furthermore, there is limited available data on how stigma varies among different subgroups, such as based on gender, race, religion, or socioeconomic status. These limitations will be considered during the interpretation of the study results and may influence the generalizability of findings to broader contexts.

## Conclusion

The stigma and discrimination faced by individuals’ with SUDs experience persist not only in the community but also within PATCs, and medical settings. This Stigmatization adversely affects the accessibility and acceptability of care, as the lack of anonymity limits the willingness of this population to seek SUD treatment. The present study aims to provide comprehensive insights into the development of appropriate strategies to reduce addiction-related stigma and discrimination at PATCs. By incorporating evidence-based practice principles, insights from people who use drugs’ experiences, and input from PATC staff, these strategies can offer valuable guidance for healthcare professionals, policymakers, and managers seeking to enhance the quality of care for individuals with a history of drug use worldwide. Furthermore, the strategies developed may serve as a blueprint for adapting interventions for patients with SUDs in various settings, including other healthcare treatment centers, clinics, and within the broader public community.

## Data Availability

Not applicable.

## Abbreviations

SUDs: substance use disorder
PATCs: public addiction treatment centers
PSAS: Perceived Stigma of Addiction Scale
PDAAT-SA: Professional’s Discrimination, Acceptance, Attitude, and Training toward Substance Abusers
SDS: Social Distance Scale
FGDs: focus group discussions
SBIRT: screening, brief intervention and referral to treatment
IDIs: in-depth interviews
NGT: nominal group technique
M: mean
SD: standard deviation
